# Supplementary material for: Patterns of Intron Gain and Loss in Fungi
Source: PLoS Biol. 2004 Nov 30;2(12):e422. doi: 10.1371/journal.pbio.0020422 (PMC532390; doi:10.1371/journal.pbio.0020422)
Supplement: Table S1 — Also available at http://genes.mit.edu/NielsenEtAl/. (4.3 MB ZIP). [file pbio.0020422.st001.zip › NielsenEtAl/html/206.html]

AN0435.1.NCU08387.1.MG03651.1.FG01520.1


```
 CLUSTAL W (1.82) Multiple Sequence Alignments - Introns Inserted


Sequence 1: MG03651.1	287 aa
Sequence 2: FG01520.1	286 aa
Sequence 3: NCU08387.1	299 aa
Sequence 4: AN0435.1	278 aa
Alignment Length: 307 aa
Number Identitical Residues: 112 aa
Alignment Score (without introns) 5682


MG03651.1 	MFSSRFNQPPPSSKMSFTKSHRASKSSSS--FSKESGISKRRHDSHRESRRRPTTATTSI
NCU08387.1	-MSSRCLDIPHSKSGSRPPKLVRSNSSLS--FAKEAGISKRRHDLRHR---RLNVATVPG
FG01520.1 	MFST--SRRHESSTKSFSKGHKASKSSAS--FSKDSGISKRKHESRHS--RRPTMTSMTS
AN0435.1  	MLTTR--RNHQSHNDSFSSRYGTSKASRSRRHARDASVASR-GPVRRR-----TPASPPD
          	 :::       * . * .     *::* *  .::::.::.*    ::      . :: . 

MG03651.1 	TA~GDES~P2--~NLTSAIVTLVVGEEQRIFAAHEHVLSHSAYFEAALR--DQFMDASSK
NCU08387.1	TV~ARAD2G~NL~NPISPIVTLLIGQESRIFAAHENVLSASPFFKNVLD--TQFSEGETK
FG01520.1 	TS~GDTT~M~GD2NMASPIVTLVVGSEQRLFAAHEDVLSNSPFFNHALR--NGYMDSASK
AN0435.1  	GS2----~-~--~STSSSVITISVGSDRRLFAAHKEILCVSPFFATACSRARSLNPSSSR
          	             .  *.::*: :*.: *:****:.:*. *.:*  .   :     . ::

MG03651.1 	RISIPDEEPEIFSSVLEYLYKGDYYPRLVHNKRKNSWELEPTTEDGRA------ESTVLH
NCU08387.1	RILLPNEEPEIFSSVLEYLYKGDYHPRLVHDRRRDTWELEESAAAGRNGNGGREAPTIYH
FG01520.1 	RISLPDEEPEIFSSVLEFLYKGDYTPRLVHNKRRNSYELETASEEQRAA----VESTVYH
AN0435.1  	RISLPEEQPEVFSCILEYLYRGDYYPRLVQNEKHNTWELENPKLEDGQT----DGATLFH
          	** :*:*:**:**.:**:**:*** ****::.:::::*** .             .*: *

MG03651.1 	RRAEGDLLKDTVIY~CAAHRYGLDELKRVALRKQGLQ1SGIQCSTILASARYAYAHTPDT
NCU08387.1	HSVDGEVLKDTVIY~CAGEKYGLEELKRIALRKQGLQ1CGRVASTILTSARYAYANTPDT
FG01520.1 	RGIDGDLLKDTVIY~CAAEKYGLDELKKISLRKQGLQ1SGIQCSTILASARYAYANTPDT
AN0435.1  	PAAGAEILRDTAVY2CAANKYGLEHLKRLSLVKQGLH~TGIQCSTILASARYAYANTPDH
          	    .::*:**.:* **..:***:.**:::* ****:  *  .****:*******:*** 

MG03651.1 	DSKLRAHYLALIIRSRSTFKKSGTMQMEMYNGGTPLFFDLFVALCNHV~DDVSTAQ2TTP
NCU08387.1	DSKLRAHYLALIIRGRNTFKRSGTMQLEMFHGGSDLFFDLFVALCNHV~DDISVAS2STQ
FG01520.1 	DSKLRAHYLALIIRSRSTFKRSGTMQLEMYNGGTQLFFDLFVALCNHV~DDISTAQ2NTP
AN0435.1  	ESKLRAHYLALIIRSRSTFKRSGTMQMEMEKGG-KLFFDLFVAMCNHM0DDLAAAA~PST
          	:*************.*.***:*****:** :**  ********:***: **::.*   : 

MG03651.1 	RSNRHFF--------
NCU08387.1	QNTQQSSCNEDGPQP
FG01520.1 	RSNRHS---------
AN0435.1  	KRSLAA---------
          	: .
```
